# Target dependence of orientation and direction selectivity of corticocortical projection neurons in the mouse V1

**DOI:** 10.3389/fncir.2013.00143

**Published:** 2013-09-23

**Authors:** Teppei Matsui, Kenichi Ohki

**Affiliations:** ^1^Department of Molecular Physiology, Graduate School of Medical Sciences, Kyushu UniversityFukuoka, Japan; ^2^CREST, Japan Science and Technology AgencyTokyo, Japan

**Keywords:** visual cortex, mouse, corticocortical connection, *in vivo* two photon imaging, axon

## Abstract

Higher order visual areas that receive input from the primary visual cortex (V1) are specialized for the processing of distinct features of visual information. However, it is still incompletely understood how this functional specialization is acquired. Here we used *in vivo* two photon calcium imaging in the mouse visual cortex to investigate whether this functional distinction exists at as early as the level of projections from V1 to two higher order visual areas, AL and LM. Specifically, we examined whether sharpness of orientation and direction selectivity and optimal spatial and temporal frequency of projection neurons from V1 to higher order visual areas match with that of target areas. We found that the V1 input to higher order visual areas were indeed functionally distinct: AL preferentially received inputs from V1 that were more orientation and direction selective and tuned for lower spatial frequency compared to projection of V1 to LM, consistent with functional differences between AL and LM. The present findings suggest that selective projections from V1 to higher order visual areas initiates parallel processing of sensory information in the visual cortical network.

## Introduction

The cerebral cortex is a hierarchically organized network that processes information in a parallel and distributed manner (Felleman and Van Essen, 1991). In the visual cortical network, information arrived at the primary visual cortex (V1) is passed to two functionally distinct cortical pathways: The dorsal pathway that consists of extrastriate cortical areas specialized for the processing visual information important for object recognition, and the ventral pathway that consists of cortical areas specialized for the processing of visual information important for spatial navigation, in primates (Ungerleider and Mishkin, 1982) and carnivores (Payne, 1993; Toyama et al., 1994). Neurons in the dorsal pathway have sharper direction selectivity and are tuned to higher temporal frequency stimuli compared to neurons in the ventral pathway that have sharper orientation selectivity and are tuned to higher spatial frequency stimuli (Maunsell and Van Essen, 1983; Albright, 1984; Desimone and Schein, 1987; Toyama et al., 1994; Pollen et al., 2002; Priebe et al., 2003). Analogous functionally distinct cortical pathways have also been found in auditory cortical pathways (Tian et al., 2001; Lomber and Malhotra, 2008).

**Figure 1:**
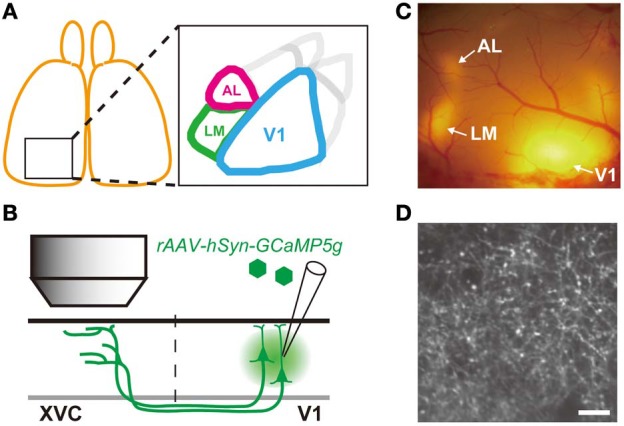
**Experimental Strategy. (A)** Schematic drawing of visual area map in the mouse visual cortex. AL and LM are the two extrastriate visual areas targeted in this study. **(B)** Schematic drawing of experimental approach. AAV containing GCaMP5g was injected into V1. Axon terminals of V1 neurons projecting to the extrastriate visual cortex (XVC) were imaged by *in vivo* two photon microscopy. **(C)** Epifluorescence TurboRFP signal observed *in vivo* through the cranial window. Patches of red fluorescence are observed in V1 (injected area) as well as in XVC as smaller patches of fluorescence. **(D)** Representative image of axon terminals expressing GCaMP5g observed *in vivo* by two photon microscopy in area LM indicated in **(C)**. Individual axonal boutons can be clearly resolved as bright varicosities. Scale bar, 10 μm.

As in primates, mouse visual cortex consists of V1 and extrastriate visual cortices that receive direct projection from V1 (Figure 1A; Wang and Burkhalter, 2007). Based on anatomical connectivity, Wang and Burkhalter suggested that these extrastriate cortices could be grouped into dorsal and ventral pathways analogous to dorsal and ventral pathways in primates and carnivores (Wang et al., 2011, 2012). In particular, two extrastriate areas, namely AL and LM, were identified as the first stage after V1 for the dorsal and ventral pathways, respectively. In line with this idea, recent *in vivo* imaging studies revealed functional distinction between visual response properties of neurons in the extrastriate areas: Neurons in AL have higher orientation/direction selectivity and are tuned to lower spatial frequency information than neurons in LM (Marshel et al., 2011). It was also reported that neurons in AL are tuned response to lower spatial frequency and higher speed stimuli than neurons in another extrastriate area PM (Andermann et al., 2011).

In the primate V1, neurons responding to distinct visual features are spatially segregated into distinct anatomical modules [e.g., cortical layers or columns; Livingstone and Hubel, 1988; but see Nassi and Callaway (2009) for existence of crosstalk between modules] each of which is connected to specific higher order visual areas to form distinct functional pathways [Livingstone and Hubel, 1988; but see Sincich and Horton (2005) for existence of crosstalk between pathways]. However, since distinct visual features are represented in a spatially intermingled manner in rodents, it is unlikely that specific connection between laminar and/or columnar modules are used to selectively route information about visual feature to different higher order visual areas. One potential mechanism to create functional specialization of extrastriate visual areas in rodents is that distinct information intermingled in V1 is selectively routed to relevant pathways with cellular level specificity. Another possibility is that information fed by V1 is not different across two pathways but is processed differently by local neuronal circuit in the target extrastriate areas to extract different features of visual information. It has been difficult to distinguish between these two possibilities due to a technical difficulty of identifying projection targets of neurons whose activity has been recorded.

Recently, genetically encoded calcium indicators have been greatly improved (Tian et al., 2009; Horikawa et al., 2010; Akerboom et al., 2012) enabling *in vivo* recording of activity from fine neuronal processes such as dendrites and axons (Petreanu et al., 2012; Xu et al., 2012). A recent study took advantage of this technology to record activity of axon terminals projecting from the mouse V1 to higher order visual areas and found difference in the optimal spatial and temporal frequencies between axon terminals projecting to AL, LM (Glickfeld et al., 2013). Since sharpness of orientation and direction selectivity also differs between neurons in AL and LM (Marshel et al., 2011), it is of great interest whether functional property of axonal projections from V1 to AL and LM differs in these two features.

In the present study, we used genetically encoded calcium indicator to observe axonal calcium activity of corticocortical projection neurons in the mouse V1 (Petreanu et al., 2012; Glickfeld et al., 2013). We infected neurons in the mouse V1 by injecting recombinant adeno-associated-virus (rAAV) carrying genetically encoded calcium indicator (GCaMP5g; Akerboom et al., 2012). We then imaged calcium activity of the axon terminals of infected V1 neurons at their projection target (Figure 1B). We found that sharpness of orientation and direction selectivity as well as optimal spatial frequency of axon terminals projecting from V1 to AL and LM were distinct and match with the functional characteristics of the neurons in the target areas.

## Materials and methods

### Animals and viral injection

Wild type C57/BL6 mice around two to three months of age were prepared for viral injection. Mice were anesthetized with an intraperitoneal injection of chloral hydrate (4 mg/g) and an intramuscular injection of xylazine (2 μg/g). After opening the scalp, a small craniotomy (~1 mm diameter) was made over the left V1 (~3 mm lateral from the midline and ~1.5 mm posterior from the lambda). A glass pipette (tip diameter, 50 μm) containing rAAV-*hSyn*-*GCaMP5g* mixed with rAAV-*CB7*-*TurboRFP* (mixed at 10:1; purchased from the University of Pennsylvania Human Gene Therapy Vector Core) were inserted to the cortex at a depth of ~400 μm. Then a small amount of virus solution (0.1–0.5 μl) was pressure injected at a rate of 0.05 μl/min using a syringe pump (SP101I, World Precision Instruments, Sarasota, FL). Imaging experiments commenced around three weeks after the injection. All experimental procedures used in this study were approved by the Animal Care and Use Committee of Kyushu University.

### *In vivo* two photon imaging

Detailed procedure for the preparation of *in vivo* two photon imaging is described elsewhere (Ohki and Reid, 2011). Briefly, anesthesia was induced with isoflurane (3%) and maintained with isoflurane (1–2% in surgery, 0.5–1% during imaging). After opening the scalp, the location and the extent of RFP expression was examined through the skull with green LED light. A custom made metal headplate was attached to the skull using dental cement (SunMediacal, Shiga, Japan), and a craniotomy (~5 mm) was made to expose the cortical surface expressing RFP. After the craniotomy, the dura was removed and exposed cortex was covered by a circular glass window (6.5 mm diameter). We often found a large patch of RFP expression in V1 surrounded by several smaller patches of RFP at the extrastriate areas (Figure 1C). This pattern of RFP expression was used to select location for calcium imaging.

*In vivo* imaging of axonal calcium activity was performed using a two photon microscope (A1RMP, Nikon, Tokyo, Japan) equipped with a X25 water immersion objective (NA1.1, Nikon). GCaMP5g was excited at 920 nm wavelength by a Ti:Sapphire laser (Mai Tai HP DeepSee, Spectra Physics). A square region of cortex 64 μm on each side (512 × 512 pixels) was imaged at 30 Hz. Depth of the imaged plane was carefully adjusted manually every 5–10 min. Image planes from the same cortical location were separated at least by 10 μm in the depth direction to avoid imaging the same axonal boutons twice. During the imaging, the level of anesthesia was adjusted by monitoring the heart rate continuously by electrocardiogram. Body temperature was maintained at 37°C by a feedback-controlled heat-pad. Silicon oil was used to prevent eyes from drying.

For each imaged region its location was identified by matching the spatial pattern of blood vessels on the cortical surface in the two-photon images and that in a macroscopic picture of RFP expression pattern. Subsequently, corresponding extrastriate visual area was assigned to each imaged region based on the pattern of RFP expression and/or retinotopic map obtained with intrinsic signal optical imaging.

### Intrinsic signal optical imaging

Mapping of cortical retinotopy by optical imaging of intrinsic signal was performed according to the method described in previous studies (Kalatsky and Stryker, 2003; Marshel et al., 2011). Briefly, prior to the optical imaging experiment, mice had either thinned skull or an implanted glass window over the left visual cortex. Throughout the imaging, mice were anesthetized by isoflurane (0.6–1.2%), and 700 nm LED light source was used to illuminate the brain. Data was collected at a frame rate of 5 Hz using a CCD camera (1000-m, Adimec, Boston, MA) controlled by an Imager3001 system (Optical Imaging Ltd., Rehovot, Israel). Obtained signal was Fourier transformed to extract the phase at the stimulus frequency (one cycle in 20 s), which can then be converted to the position in the visual space.

### Visual stimulation

Visual stimuli were presented on a LCD display using a desktop computer running PsychoPy (Peirce, 2009) or a custom made software written in Visual Basic (Microsoft). For mapping of orientation and direction preferences, a drifting square-wave grating [100% contrast; 0.04 cycles per degree (cpd); 2 Hz] tilted at one of four orientations in 45° steps moving in one of two directions orthogonal to the orientation (yielding total of eight directions of motion in 45° steps) was presented. Each stimulus started with a blank period of uniform gray (4 s) followed by the same period of visual stimulation. Each condition was repeated 10–20 times. For mapping of spatial frequency (SF) and temporal frequency (TF) tunings, drifting sine-wave gratings (100% contrast) were used. For SF mapping experiments, sine-wave gratings having six SF between 0.01 and 0.4 cpd and drifting at 2 Hz were used. For TF mapping experiments, sine-wave gratings having 0.04 cpd and drifting at 5 different TF between 0.5 and 8 Hz were used. Each stimulus started with a blank period of uniform gray (4 s) followed by the same period of visual stimulation during vertical and horizontal gratings were presented for 1 s for each of four directions (0°, 180°, 90°, and 270° in order). Each condition was presented 10–20 times in pseudorandom orders. In SF and TF mapping experiments, both SF and TF stimuli were tested at each imaged plane. Stimuli for mapping retinotopy by intrinsic signal optical imaging were adapted from a previous study (Kalatsky and Stryker, 2003). A thin flashing white bar on a black screen was continuously moved in a horizontal or vertical direction at a constant speed of 20 s/cycle. Each run lasted 320 s (16 cycles).

### Analysis of two photon calcium imaging data

**Figure 2:**
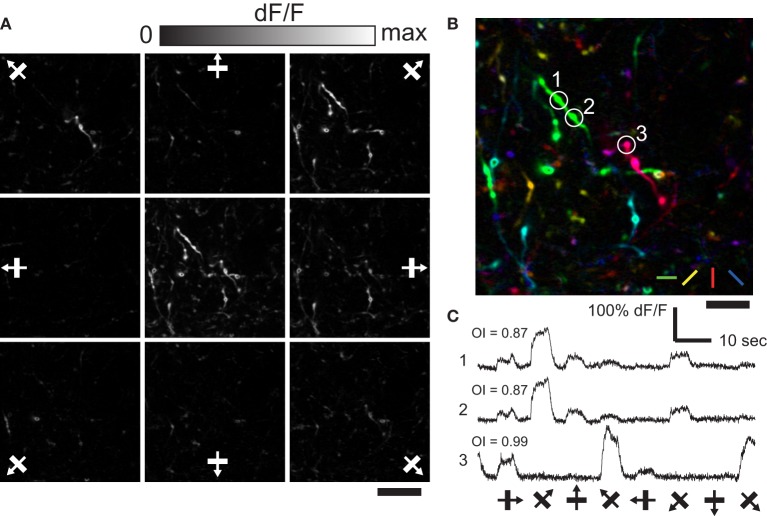
**Orientation and direction selective response of axonal boutons in LM. (A)** Representative single condition ΔF/F maps for eight directions. Center panel shows max ΔF/F. Scale bar, 20 μm. **(B)** Color-coded map created from **(A)**. Scale bar, 10 μm. **(C)** Representative time courses (average of 10 trials) of three axonal boutons in **(B)**. Note that boutons belonging to the same axon fiber (1 and 2), show very similar time courses.

All the analyses were performed using custom software written in Matlab (MathWorks, Natick, MA). Acquired images were first realigned by maximizing the correlation across frames. Axon terminals were automatically identified by template matching with a circular template using time averaged image. Time courses of individual axon terminals (boutons) were extracted by summing pixel values within the contours of axonal boutons. Slow drift of the baseline signal over minutes was removed by a low-cut filter (Gaussian, cutoff, 1.6 min) and high-frequency noise was removed by a high-cut filter (first-order Butterworth, cutoff, 1.6 s). Visually responsive axonal boutons were defined by ΔF/F > 0.15 and by one way analysis of variance (*p* < 0.01) across blanks and stimulus periods. The response to each orientation was defined as the mean of the responses to two drifting gratings moving at opposing directions orthogonal to the orientation (e.g., response to 0° orientation was obtained by averaging responses to two gratings moving orthogonal to the 0° orientation, i.e., 90° and 270° directions). Of these boutons, boutons selectively responding to stimulus conditions were defined by one way analysis of variance (*p* < 0.01) across stimulus conditions (four orientations for orientation mapping experiments, and six SF and five TF for SF and TF mapping experiments, respectively). Preferred direction for pixel based direction map (Figure 2B) was calculated by vector averaging (Swindale et al., 1987). For the orientation and direction preference analyses Orientation Index (OI) was calculated by the formula: *OI* = 1 − *R*_ortho_ / *R*_pref_, where *R*_pref_ is the response to the preferred orientation and the *R*_ortho_ is the response to the orientation orthogonal to the preferred orientation. Direction Index (DI) was calculated by the formula: *DI* = 1 − *R*_null_ / *R*_pref_ (Mikami et al., 1986), where *R*_pref_ is the response to the preferred direction and the *R*_null_ is the response to the direction opposite to the preferred direction. For the SF and TF analyses, difference of Gaussian (DOG) was fitted to each axonal bouton's response (Hawken and Parker, 1987). Preferred SF (or TF) for each axonal bouton was then defined by SF (or TF) at the maximum of the fitted DOG. The preferred SF (or TF) was rounded to the maximum or the minimum value of the tested stimulus parameter, when it fell outside of these values. All statistical testing was performed using Statistics Toolbox of Matlab (MathWorks).

## Results

### *In vivo* imaging of axonal activity of V1 projection neurons

Mice were injected with a mixture of rAAV encoding GCaMP5g and rAAV encoding TurboRFP into V1 resulting in a large patch of red (and green) fluorescence in V1 that was surrounded by smaller patches of fluorescence in the extrastriate areas (Figure 1C). Consistent with previous anatomical tracing study, large RFP patches were found in areas AL and LM (Wang and Burkhalter, 2007). At these extrastriate RFP patches, axonal fibers and boutons of V1 projection neurons expressing GCaMP5g (and TurboRFP) could be detected *in vivo* at the cortical depth from ~20 μm up to ~500 μm (Figure 1D). Large calcium transients in response to visual stimulation could be observed from axonal boutons expressing GCaMP5 Supplementary Movie 1). We first characterized orientation and direction preference of V1 projection neurons by analyzing calcium activity of these axonal boutons.

Many axonal boutons in AL selectively responded to a presentation of particular orientation of drifting square gratings (Figure 2A; *n* = 108 visually responsive boutons out of 476 boutons identified. see Materials and Methods). Typically, orientation preference of nearby boutons were different (Figure 2B), however boutons belonging to the same axonal fiber showed matched orientation preference as well as closely matched time courses of calcium responses (Figure 2C; see Petreanu et al., 2012). Similar spatial organization for orientation preference of axonal boutons was found in LM (data not shown). These results show spatially intermingled pattern of V1 input for orientation information in AL and LM. Moreover, the fact that we could clearly detect the difference in the orientation preference of nearby axonal boutons demonstrates the reliability of our recording from individual axonal boutons.

### Sharpness of orientation and direction selectivity of V1 axons differs in AL and LM

**Figure 3:**
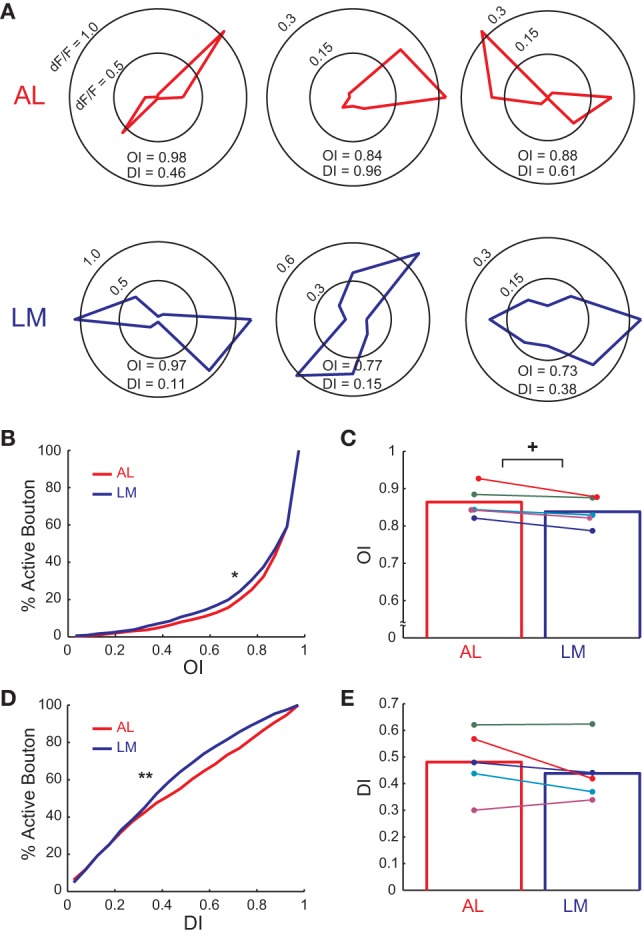
**Orientation and direction tuning property of axonal boutons in AL and LM. (A)** Polar plots showing responses (ΔF/F) to eight directions for example axonal boutons in AL and LM. **(B)** Cumulative distribution of OI of axonal boutons in AL and LM. Mean OI across all responsive boutons = 0.851 and 0.829 in AL and LM, respectively. ^*^, *P* < 0.006. **(C)** Mean OI in AL and LM for each animal. Mean OI across animals = 0.864 and 0.838 in AL and LM, respectively. Each pair of colored dots indicates data from one animal. Bar graph shows mean of all the animals. ^+^, *P* < 0.04. **(D)** Cumulative distribution of DI of axonal boutons in AL and LM. Mean DI across all boutons = 0.463 and 0.418 for AL and LM, respectively. ^**^, *P* < 0.0001. **(E)** Mean DI in AL and LM for each animal. Each pair of colored dots indicates data from one animal. Mean DI across animals = 0.481 and 0.438 for AL and LM, respectively. Bar graph shows mean of all the animals.

Next we investigated difference in orientation and direction selectivity of axonal boutons in AL and LM. Individual axonal boutons showed highly tuned response to drifting gratings presented at different orientations and directions in both AL and LM (Figure 3A). Of all the visually responsive axonal boutons in AL and LM (1250 and 1630 boutons in AL and LM, respectively), 1068 (85%) and 1490 (91%) responded selectively to orientation and direction of drifting gratings in AL and LM, respectively. Cumulative distribution of OI for all visually responsive axonal boutons in AL collected from five mice was significantly shifted toward higher values than that in LM (*P* < 0.006, Kolmogorov-Smirnov test; Figure 3B), indicating that V1 neurons projecting to AL were more orientation selective than those projecting to LM. To test for consistency across animals, we also compared mean OI of axonal boutons in AL and LM for each animal. Consistent with the cumulative data, mean OI in each animal was higher in AL than in LM for all the animals and the difference was statistically significant (*P* < 0.04, *n* = 6, sign-rank test; Figure 3C).

Next we examined the difference in direction selectivity by calculating DI for the same set of boutons in AL and LM. Cumulative distribution of DI for axonal boutons in AL pooled from all the animals was significantly shifted toward higher values than that in LM (*P* < 0.0001, Kolmogorov-Smirnov test; Figure 3D), indicating that V1 neurons projecting to AL were more direction selective than those projecting to LM. Though not statistically significant, the difference in DI across two areas was consistently observed across animals as the mean DI was larger in AL than in LM (Figure 3E). When the analysis was restricted to selectively responding boutons, four out of five animals had higher mean DI in AL than in LM (data not shown). Taken together, the functional difference of axonal activity in AL and LM found here closely matches with a previous finding which reported sharper orientation and direction selectivity for neurons in AL than neurons in LM (Marshel et al., 2011).

### Spatial and temporal frequency preference of V1 axons differs in AL and LM

**Figure 4:**
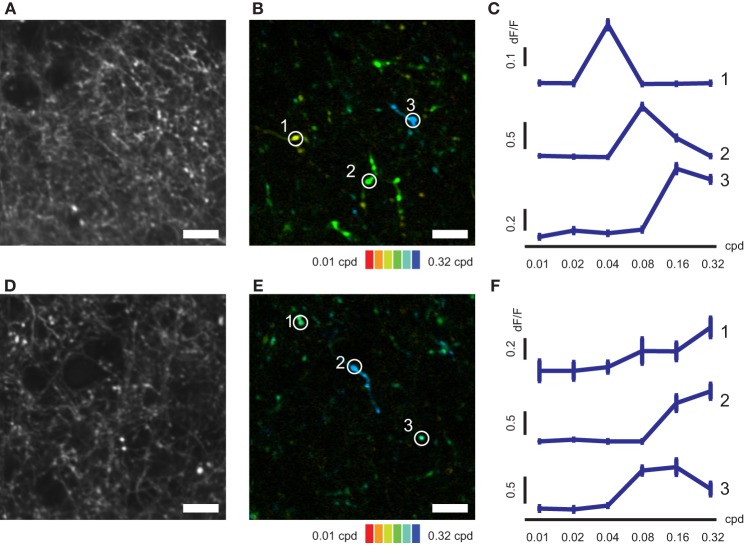
**SF-tuned responses of axonal boutons in AL and LM. (A)** Representative FOV in LM. **(B)** Color-coded map of preferred SF of axonal boutons shown in **(A)**. **(C)** Representative SF tuning curves for three axonal boutons indicated by circles in **(B)**. **(D)** Representative FOV in AL. **(E)** Color-coded map of preferred SF of axonal boutons shown in **(D)**. **(F)** Representative SF tuning curves for three axonal boutons indicated by circles in **(E)**. Error bars indicate s.e.m. Scale bars, 10 μm.

**Figure 5:**
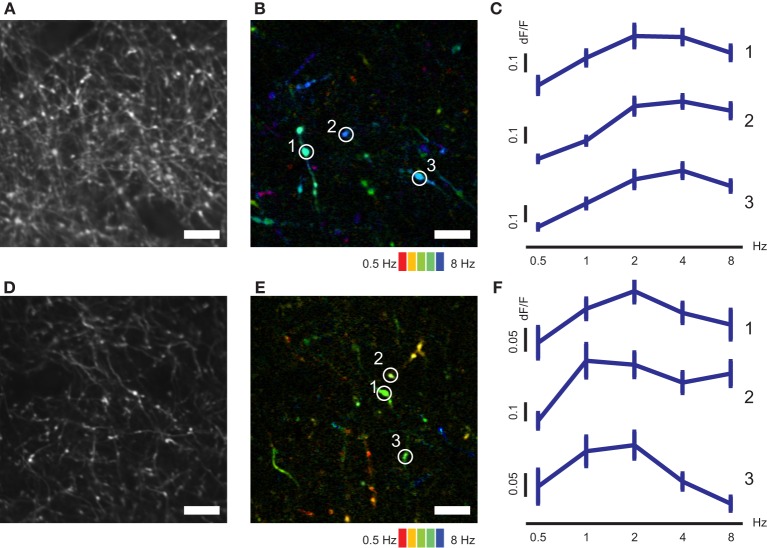
**TF–tuned responses of axonal boutons in AL and LM. (A)** Representative FOV in LM. **(B)** Color-coded map of preferred TF of axonal boutons shown in **(A)**. **(C)** Representative TF tuning curves for three axonal boutons indicated by circles in **(B)**. **(D)** Representative FOV in AL. **(E)** Color-coded map of preferred TF of axonal boutons shown in **(D)**. **(F)** Representative TF tuning curves for three axonal boutons indicated by circles in **(E)**. Error bars indicate s.e.m. Scale bars, 10 μm.

We next conducted mapping of SF and TF tunings of V1 axons in AL and LM (Figures 4, 5). Individual axonal boutons showed variety of SF and TF tuning in both AL and LM (SF, Figures 4B,E; TF, Figures 5B,E). SF and TF tuning curves obtained for individual axonal boutons were similar to those reported for neurons in the mouse V1 (SF, Figures 4C,F; TF, Figures 5C,F; Niell and Stryker, 2008). As in the case of orientation and direction preference, visually responsive axonal boutons having various SF and TF tuning were spatially intermingled both in AL and LM without any apparent local clustering according to SF or TF preference.

**Figure 6:**
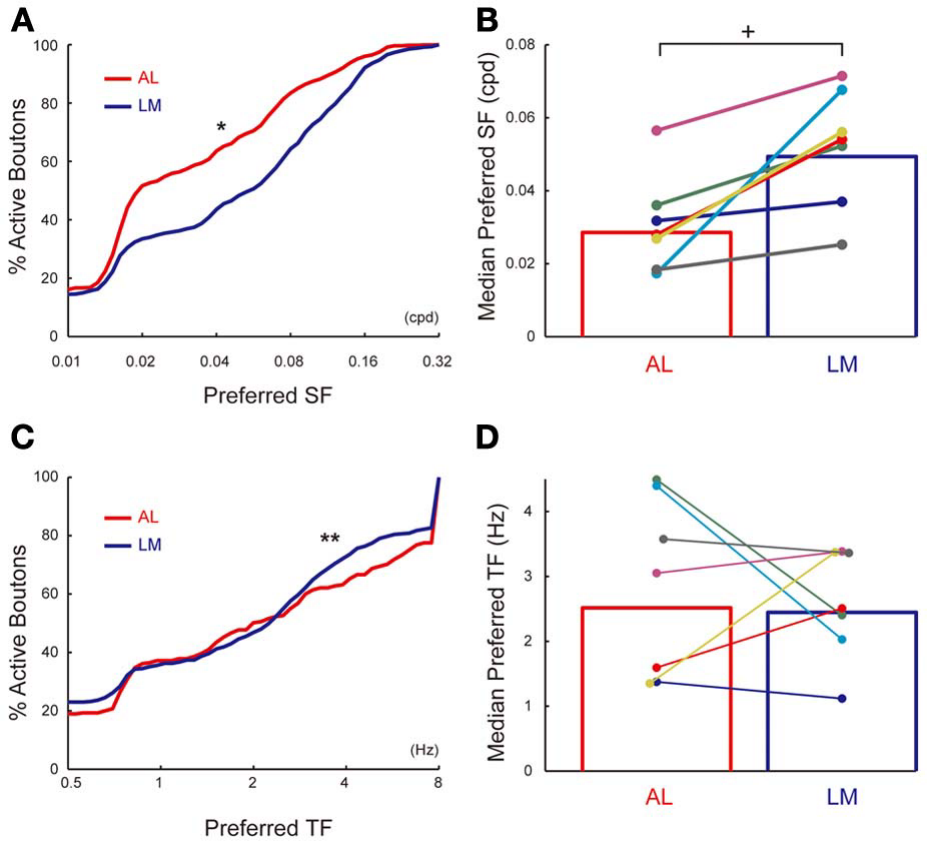
**SF and TF tuning properties of axonal boutons in AL and LM. (A)** Cumulative distributions of preferred SF in AL and LM. Mean of preferred SF across all boutons = 0.031 and 0.048 cpd for AL and LM, respectively. ^*^, *P* < 0.001. **(B)** Mean preferred SF in AL and LM for each animal. Each pair of colored dots indicates data from one animal. Mean of preferred SF across all animals = 0.029 and 0.049 cpd for AL and LM, respectively. Bar graph shows mean of all the animals. ^+^, *P* < 0.01. **(C)** Cumulative distributions of preferred TF in AL and LM [same set of boutons as in **(A)**]. Mean of preferred TF across all boutons = 2.1 and 1.9 Hz for AL and LM, respectively. ^**^, *P* < 0.03. **(D)** Mean preferred TF in AL and LM for each animal. Mean of preferred TF across all animals = 2.5 and 2.4 Hz for AL and LM, respectively. Each pair of colored dots indicates data from one animal. Bar graph shows mean of all the animals.

Of all the visually responsive axonal boutons in AL and LM (835 and 1236 boutons in AL and LM, respectively), 796 (95%) and 1178 (95%) responded selectively to drifting gratings presented at different spatial frequencies in AL and LM, respectively, and were further analyzed. Cumulative distribution of preferred SF (see Materials and Methods for obtaining preferred SF) for population of axonal boutons in LM pooled from seven mice was shifted significantly toward higher SF compared to that in AL (*P* < 0.0001, Kolmogorov-Smirnov test; Figure 6A). Consistently, mean value for the preferred SF in LM calculated separately for each animal was significantly higher than that in AL (*P* < 0.008, *n* = 7, sign-rank test; Figure 6B). Finally, we examined the difference in TF tuning of axonal boutons in AL and LM. Of all the visually responsive axonal boutons in AL and LM (438 and 685 boutons in AL and LM, respectively), 285 (65%) and 444 (65%) responded selectively to drifting gratings presented at different temporal frequencies in AL and LM, respectively, and were further analyzed. The cumulative distribution of the preferred TF pooled from all the animals was larger in AL than in LM (*P* < 0.03, Kolmogorov-Smirnov test; Figure 6C). Although not significant, mean preferred TF across animals was also larger in AL than in LM (*P* = 0.4, *n* = 7, sign-rank test; Figure 6D). These differences of the axonal boutons in AL and LM found here for SF tuning, and to a weaker extent for TF tuning, are consistent with a recent report (Glickfeld et al., 2013). As in the case for orientation and direction selectivity, the difference in the SF preference of axons in AL and LM matches with that of the reported response properties of neurons in AL and LM (Marshel et al., 2011). Taken together, the present results suggest that the feedforward corticocortical projections from V1 to AL and LM are functionally distinct in a way that matches with the reported functional difference between neurons in AL and LM.

## Discussions

In the present study, by using *in vivo* two photon calcium imaging of axonal activity, we characterized difference in the visual response properties of corticocortical projection neurons in the mouse V1. Response properties of the corticocortical projection neurons in V1 were significantly different depending on their target extrastriate areas: Axonal boutons of V1 neurons projecting to AL had sharper of orientation and direction selectivity and responded optimally for lower spatial frequency stimuli compared to axonal boutons projecting to LM. These differences in the visual response properties of V1 projection neurons in AL and LM were largely consistent with the reported response properties of the neurons in AL and LM. Therefore, the present results support the notion that functional specialization in the higher order cortical areas is created by selective projections of functionally distinct neurons from the upstream cortical areas.

### Advantages of axonal calcium imaging compared to other techniques for studying corticocortical projection neurons

Previous electrophysiological studies have used techniques such as antidromic electrical stimulation [Movshon and Newsome (1996) among many others], optogenetic stimulation (Lima et al., 2009) and fluorescent dye filling by electroporation (Igarashi et al., 2012) to identify projection target of neurons whose response properties were characterized electrophysiologically. However, these techniques require significant labor to collect a large number of samples. Indeed, out of 786 neurons recorded in V1, only 12 could be identified as MT projecting neurons by means of antidromic electrical stimulation (Movshon and Newsome, 1996). The present imaging-based approach possesses several advantages compared to these previous approaches. First, it is technically straight forward and does not require complicated experimental steps such as identification of antidromically stimulated neurons or single neuron electroporation. Second, and more importantly, the present approach enables collection of many samples of projection neurons from multiple target areas from individual animals. This second advantage was critical in revealing subtle but statistically significant differences between two overlapping distribution of the neuronal response properties.

Several groups have used combination of retrograde neuronal tracing and *in vivo* two photon calcium imaging of labeled neuronal somata to study corticocortical projection neurons (Sato and Svoboda, 2010; Osakada et al., 2011; Jarosiewicks et al., 2012; Chen et al., 2013). Although this approach is promising and complementary to axonal calcium imaging, it is necessary to use multiple colors to label neurons projecting to multiple areas in one animal (Jarosiewicks et al., 2012). Nevertheless, it is of great importance to see whether experiments using retrograde tracers reach the same conclusions as that reached by axonal calcium imaging.

It should be noted that genetically encoded calcium indicator used in the present study (GCaMP5g) is not capable of reporting single action potential reliably (Akerboom et al., 2012). Hence, it is not clear whether large portion of axon terminals that were non-responsive to visual stimulation were indeed non-visually driven V1 neurons (Keller et al., 2012) or their response simply did not reach sensitivity limit of GCaMP5g. In addition, slow time course of GCaMP5g prohibited us from analyzing action potential synchrony of projection neurons which may be important for efficient corticocortical spike transmission (Fries, 2009). Development of more sensitive genetically encoded calcium or voltage indicators with fast kinetics will be critical to resolve these problems (Jin et al., 2012).

### Comparison with previous studies

Functional specialization of extrastriate visual areas in the mouse has been reported by previous imaging studies (Andermann et al., 2011; Marshel et al., 2011; Roth et al., 2012). A recent study (Glickfeld et al., 2013) that used similar approach to ours revealed that the difference in the SF and TF tunings among extrastriate areas (AL, LM, and PM) could be accounted for by difference in the SF and TF tuning of V1 neurons projecting to each of these areas. The present study confirms and adds to this result by showing that the difference in the projection neuron can also contribute to the difference in orientation and direction selectivity of neurons in the target area. Since neurons in the dorsal lateral geniculate nucleus are selective for SF and TF (Grubb and Thompson, 2003), target-dependence of the optimal SF and TF of corticocortical projections may be explained by mechanism similar to parallel visual pathways found in primates that relay functionally distinct visual information from subcortical areas (e.g., retina or LGN) to primary and extrastriate visual cortices (Livingstone and Hubel, 1988). Although, unlike primates, neurons preferring various SF and TF are not spatially clustered in mice, parallel functional channels similar to primates may still exist in a spatially intermingled manner (Gao et al., 2010). However, since orientation and direction selectivity are most pronounced in the cortex and rare in LGN (Marshel et al., 2012; Piscopo et al., 2013), similar mechanism based on parallel pathways from subcortical to cortical areas may not be sufficient in these cases. Moreover, SF preference and orientation selectivity are not systematically related in mouse V1 neurons (Gao et al., 2010), hence a mechanism that produce difference in the SF and TF preference of V1 projection neurons cannot explain that of orientation and direction selectivity. Orientation and direction selectivity are related to specifically connected local neuronal circuits in the neocortex (Yoshimura et al., 2005; Yu et al., 2009; Ko et al., 2011). Thus, there may be some common factor that links the specifically connected local neocortical circuit and the target-dependence of orientation and direction selectivity of corticocortical projection neurons.

Differences in the tuning properties of corticocortical projections from V1 to AL and LM are largely consistent with the functional differences of dorsal/ventral pathways expected from the well-studied dorsal/ventral pathways of macaques (Ungerleider and Mishkin, 1982; Maunsell and Van Essen, 1983; Desimone and Schein, 1987; Pollen et al., 2002; Priebe et al., 2003). Axonal boutons in AL that belongs to the putative dorsal pathway in mice had sharper direction selectivity as well as lower optimal SF compared orientation selectivity that was sharper in AL than in LM. Since neurons in AL also have been shown to have sharper orientation tuning compared to neurons in LM (Marshel et al., 2011), these results may suggest across species difference in the dorsal/ventral pathways of macaques and mice.

Sharpness of orientation and direction selectivity of axon terminals observed in the present study was somewhat higher than that of mouse V1 neurons reported previously (Niell and Stryker, 2008; Andermann et al., 2011; Marshel et al., 2011; Roth et al., 2012). The difference found here may be attributed to inability of GCaMP5g to report single action potentials reliably. For GCaMP3, sensitivity for reporting action potentials in axon terminal (Petreanu et al., 2012) was lower than that in the soma (Tian et al., 2009). Lower sensitivity to action potentials in axon terminals may truncate calcium response to low level spiking activity in the axon terminal but not in the soma, hence resulting in higher orientation and direction selectivity in the axon terminal compared to that in the soma.

### Other mechanisms for creating functional specialization in the extrastriate areas

Although several studies including the present one converge to support the presence of selective routing of information between the primary and higher order sensory areas (Sato and Svoboda, 2010; Jarosiewicks et al., 2012; Glickfeld et al., 2013), other mechanisms may also contribute to shape response properties of higher order sensory neurons. Complex dendritic computation is likely to shape response properties of sensory neurons (Jia et al., 2010), and it is unclear whether such intracellular processing works to enhance or attenuate functional differences of V1 projection neurons. It should also be noted that a subset of V1 projections is known to target interneurons in the higher order visual area (Gonchar and Burkhalter, 2003). This feedforward inhibitory circuit could also contribute to shape response properties of excitatory neurons within the extrastriate areas. Nevertheless, while these additional mechanisms may work to fine-tune the response property of neurons within each higher order cortical area, target dependent functional projection from lower cortical area is likely to work as the seed for generating functional specialization in higher order cortical areas.

## Author contributions

Teppei Matsui and Kenichi Ohki conceived the study. Teppei Matsui performed experiments, analyses. Teppei Matsui and Kenichi Ohki wrote the manuscript.

## Conflict of interest statement

The authors declare that the research was conducted in the absence of any commercial or financial relationships that could be construed as a potential conflict of interest.

## Supplementary material

Supplementary Movie 1 **Representative axonal calcium activity in response to visual stimulation (x8 speed)**.
